# Overexpression of a High-Affinity Nitrate Transporter *OsNRT2.1* Increases Yield and Manganese Accumulation in Rice Under Alternating Wet and Dry Condition

**DOI:** 10.3389/fpls.2018.01192

**Published:** 2018-08-15

**Authors:** Bingbing Luo, Jingguang Chen, Longlong Zhu, Shuhua Liu, Bin Li, Hong Lu, Guoyou Ye, Guohua Xu, Xiaorong Fan

**Affiliations:** ^1^State Key Laboratory of Crop Genetics and Germplasm Enhancement, Nanjing, China; ^2^Key Laboratory of Plant Nutrition and Fertilization in Low-Middle Reaches of the Yangtze River, Ministry of Agriculture, Nanjing Agricultural University, Nanjing, China; ^3^CAAS-IRRI Joint Laboratory for Genomics-Assisted Germplasm Enhancement, Agricultural Genomics Institute in Shenzhen, Chinese Academy of Agricultural Sciences, Shenzhen, China

**Keywords:** rice, *OsNRT2.1*, manganese uptake, yield, nitrate

## Abstract

Nitrate and manganese (Mn) are necessary elements for the growth and development of rice in paddy soil. Under physiological conditions, we previously reported that the uptake of Mn in roots can be improved by the addition of external nitrate but not ammonium. To investigate the mechanism(s) of this phenotype, we produced plant lines overexpressing *OsNRT2.1* and assessed Mn uptake under alternating wet and dry (AWD) and waterlogged (WL) conditions. Under AWD condition, we observed a 31% reduction in grain yields of wild type (WT) plants compared to WL condition. Interestingly, the overexpression of *OsNRT2.1* could recover this loss, as *OsNRT2.1* transgenic lines displayed higher grain yields than WT plants. We also observed 60% higher grain Mn in the transgenic lines in AWD condition and approximately 30% higher Mn in the grain of transgenic lines in WL condition. We further found that the overexpression of *OsNRT2.1* did not alter Mg and Fe in the seeds in either growth condition. The reasons for the increased Mn content in *OsNRT2.1* transgenic seeds in AWD condition could be explained by the elevated expression of *OsNRAMP* family genes including *OsNRAMP3, OsNRAMP5*, and *OsNRAMP6* in node I, the panicle-neck, and the flag leaves. The mechanism(s) underpinning the upregulation of these genes requires further investigation. Taken together, our results provide a new function of *OsNRT2.1* in improving rice yields and grain Mn accumulation during water-saving cultivation patterns. This represents a new strategy for maintaining yield and improving food quality in a sustainable agricultural system.

## Introduction

Trace elements play a vital role in plant growth and development (Yan et al., 2006). All organisms require trace levels of manganese (Mn) for survival due to its necessity during plant metabolism and its participation in several important pathways (Socha and Guerinot, 2014) including the oxygen-evolving complex (OEX) of photosystem II (PS II). In addition, Mn plays an important role during phosphoenolpyruvate carboxykinase activation and liquid metabolism (Dziwornu et al., 2018). Thus, it is required for photosynthesis indirectly by repressing thylakoid synthesis. In addition, manganese superoxide dismutase (MnSOD) is the major mitochondrial antioxidant defense enzyme (Shen, 2015) and Mn is a co-factor/activator of many enzymes involved in the catalysis of oxidation reduction, decarboxylation and hydrolytic reactions (Marschner, 1995; Xu et al., 2007).

Mn deficiency is a global problem in agriculture (Hebbern et al., 2005). Mn deficient plants are more vulnerable to cold stress and infections by pathogens, leading to decreased crop yields (Marschner, 1995; Hebbern et al., 2005). Addressing this issue is problematic as Mn^2+^ rapidly oxidizes when supplemented into fertilizers. In this regard, further knowledge of molecular mechanisms that can enhance Mn delivery are required. Several Mn transporters contribute to the uptake, transport and maintenance of Mn homeostasis in plants. The *NRAMP* family was shown to participate in Mn transport during early plant discoveries. In *Arabidopsis, AtNRAMP1* localizes to the plasma membrane and displays root-specific expression where its function is to coordinate the absorption of Mn from soil (Cailliatte et al., 2010). *OsNRAMP5* is mainly involved in Mn uptake and accumulation in rice and its silencing significantly reduces Mn accumulation in shoots (Yang et al., 2014). *OsNRAMP3* is expressed in the node and regulates Mn transport and tissue distribution in response to environmental changes (Yamaji et al., 2013a). *OsNRAMP6* distributes to the plasma membrane and transports Mn and Fe, maintaining their balance in cells (Peris-Peris et al., 2017). In rice, Mn homeostasis is controlled by the *YSL2/6* gene. *OsYSL2* can promote the long-distance transport of Mn (Koike et al., 2004; Ishimaru et al., 2010). *OsYSL6* belongs to the Mn-nicotianamine (NA) transporter family and is required for the detoxification of high concentrations of Mn (Sasaki et al., 2011). In addition, the CAX proteins belong to the Ca^2+^/cation antiporter (CaCA) superfamily (Emery et al., 2012) and are potentially involved in Mn^2+^/H^+^ exchange to export Mn from the cytosol (Connorton et al., 2012).

Nitrogen (N) is an essential element for plant growth and development, especially for crops. Generally, N is absorbed by plants in the form of ammonium (NH_4_^+^) and nitrate (NO_3_^-^), but nitrate easily dissolves in water and is therefore lost to the environment (Jin et al., 2015). Roots acquire NO_3_^-^ via transporters distributed throughout the whole plant (Xu et al., 2012). Plants adapt to the differing NO_3_^-^ concentrations in soil by exploiting two forms of NO_3_^-^ uptake, including low-affinity transporters (*NRT1/NPF*) and high-affinity NO_3_^-^ transporters (*NRT2*) (Crawford and Glass, 1998). Particularly for rice plants, we previously identified a high-affinity NO_3_^-^ transport system. The *OsNRT2* gene family was found to play an important role during N uptake and translocation, requiring their partner protein *NAR2* to perform this function, besides *OsNRT2.3b* (Tang et al., 2012; Xu et al., 2012; Chen et al., 2016b; Chen Z.C. et al., 2017; Fan et al., 2016; Chen J.G. et al., 2017).

Simultaneously, Mn can influence NO_3_^-^ reductase activity and is associated with photosynthesis in plants (Botrill et al., 1970; Gong et al., 2011). Mn also influences N metabolism and regulates protein synthesis (Jiang, 2006). Studies have shown that the arabidopsis *chl1-5* mutant lines display reduced NO_3_^-^ uptake and a loss of *AtIRT1* expression, which is responsible for Cd uptake into root cells (Muños et al., 2004; Lux et al., 2011). Fe deficiency was also shown to inhibit N metabolism in the roots and leaves of cucumber plants (Borlotti et al., 2012). These effects suggest that NO_3_^-^ influences the uptake of trace elements in plants. In this study, we hypothesized that a close relationship between N and Mn in plants exists. We used transgenic rice over-expressing *OsNRT2.1* to examine how the different forms of N influence Mn uptake and accumulation in grain.

## Materials and Methods

### Plant Materials and Growth Conditions

We amplified the *OsNRT2.1* (AB008519) ORF (primers are displayed in **Supplementary Table S1**) using cDNA obtained from *Oryza sativa* L. ssp. Japonica cv. Nipponbare. PCR products were cloned into the pMD19-T vector (TaKaRa Biotechnology, Dalian, China) and the expression vector pTCK303 containing a ubiquitin promoter. Positive clones were verified by restriction digest analysis and DNA sequencing. Next, the binary vector pUbiquitin-*OsNRT2.1* was introduced into A. tumefaciens (strain EHA105), which was used to transform the rice embryonic callus as previously described (Ai et al., 2009). Hygromycin-resistant T0 generation transgenic rice plants were transplanted to soil and grown to obtain seeds in fields (Tang et al., 2012). Three independent T4 generation lines overexpressing *OsNRT2.1* were used for further experiments.

**FIGURE 1:**
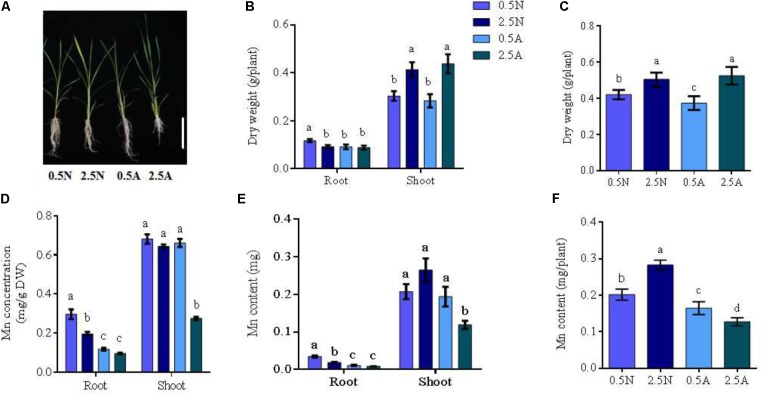
Absorption of Mn elements under different N treatments; **(A)** the phenotype of wild type (WT) rice in different N treatments; **(B)** Root and shoot dry weight; **(C)** the dry weight of whole rice plants; **(D)** Mn concentrations; **(E)** Mn content in roots or shoots; **(F)** the Mn content in the whole plant. DW, dry weight; 0.5/2.5N: 0.5/2.5 mM NO_3_^-^ as a nitrogen source; 0.5/2.5A: 0.5 mM/2.5 mM NH_4_^+^ as a nitrogen source (*n* = 4 plants). Different letters indicate a significant difference between N treatments (*P* < 0.05, one-way ANOVA). Bars = 3 cm.

**FIGURE 2:**
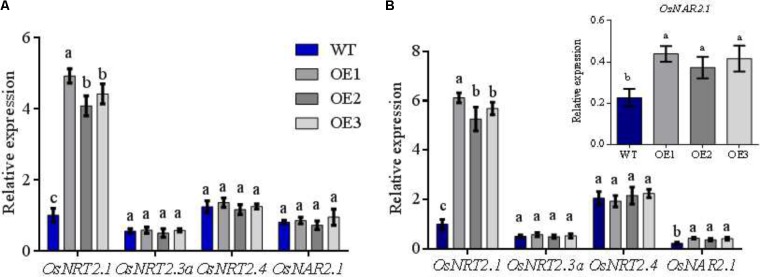
Expression of *OsNRT2s* and *OsNAR2.1* in transgenic lines. WT and transgenic lines seedlings were grown in the IRRI nutrient solution containing 1 mM NH_4_^+^ for 1 month and then treated with 0.5 mM NH_4_^+^/NO_3_^-^ for 2 weeks. Extraction of total RNA from roots of WT and transgenic lines and qRT-PCR under **(A)** 0.5 mM NH_4_^+^, **(B)** 0.5 mM NO_3_^-^. WT, wild type; OE1/2/3 was performed for the three *OsNRT2.1* transgenic lines, as below. Error bars: standard error (*n* = 4 plants). Different letters indicate a significant difference between transgenic and WT lines (*P* < 0.05, one-way ANOVA).

Firstly, rice seedlings were selected and cultured in 1 mM (NH_4_)_2_SO_4_ as the main source of N in nutrient solution (pH 5.5) for 1 month. Other elements and trace elements were supplied in IRRI (International Rice Research Institute) nutrient solution containing 0.35 mM K_2_SO_4_, 0.3 mM KH_2_PO_4_, 1 mM MgSO_4_, 1 mM CaCl_2_, 0.5 mM Na_2_SiO_3_, 20 μM H_3_BO_3_, 9 μM MnCl_2_, 20 μM EDTA-Fe, 0.77 μM ZnSO_4_, 0.32 μM CuSO_4_, and 0.39 μM (NH_4_)_6_Mo_7_O_24_. Rice were planted in a growth room (Thermoline Scientific Equipment Pty. Ltd., Smithfield, NSW, Australia) at 30°C during the day and 22°C at night with 16-h light/8-h of darkness. The light intensity was 400 μmol m^-2^ s^-1^ and the relative humidity was 65–70%. Wild type (WT) rice were then transferred to 0.25 or 1.25 mmol/L Ca(NO_3_)_2_ and 0.25 or 1.25 mmol/L (NH_4_)_2_SO_4_ nutrient solution, respectively, for 2 weeks (**Figure 1**). In **Figures 2 and 4**, WT and overexpression lines were transferred to 0.5 mM NH_4_^+^/NO_3_^-^ nutrient solution for 2 weeks. For each line and treatment, four biological repeats were performed.

In field experiments, rice were planted in Nanjing, Jiangsu; a subtropical monsoon climate zone. The characteristics of the soil and N supply were as previously described (Chen et al., 2016b). For waterlogged (WL) treatment, rice fields were watered daily to maintain WT and transgenic lines in a flooded state. For alternating wet and dry treatment (AWD), lines were planted into fields and watered for a week, to keep the soil moist.

#### Southern Blot Analysis

Transgene cope numbers were identified by southern blot analysis. Briefly, genomic DNA was extracted from the leaves of WT and transgenic lines and digested with HindIII and EcoRI. Digested DNA was separated on 1% (w/v) agarose gels, transferred to a Hybond-N^+^ nylon membrane and hybridized using the hygromycin-resistant gene.

### RNA Extraction and qPCR Analysis

Total RNA was extracted from 100 mg of tissue using TRIzol (Invitrogen, Carlsbad, CA, United States). Total RNA concentrations were assessed by UV spectrophotometry (Eppendorf, Bio-photometer, Germany). RNA (2 μg) was reverse transcribed into cDNA using HiScript Reverse Transcriptase (Vazyme, Nanjing, China) according to the manufacturers protocol. Four biological repeats were performed for each qPCR reaction, using *OsActin* as a reference gene. Primers were designed to detect *OsNRT2.1, OsNRT2.3a, OsNRT2.4, OsNAR2.1, OsNRAMP3, OsNRAMP5, OsNRAMP6, OsIRT1*, and *OsMGT1* and are listed in **Supplementary Table S2**. PCR amplification was performed using SYBR qPCR Master Mix (Vazyme, Nanjing, China). PCR reactions were performed under the following parameters: 95°C for 30 s, followed by 40 cycles of 95°C for 10 s, 60°C for 30 s, and 72°C for 10 s.

### Determination of the ^15^N-NH_4_^+^/NO_3_^-^ Influx Rate in Different Rice Lines

Rice seedlings of WT and *OsNRT2.1* transgenic rice plants were planted in IRRI solution containing 1 mM NH_4_^+^ for 2 weeks and N starved for 3 days. Plants were first transferred into 0.1 mM CaSO_4_ for 1 min, then to complete nutrient solution containing either 0.5 mM ^15^NH_4_^+^ or 0.5 mM ^15^NO_3_^-^ (atom% ^15^N: 99%) for 5 min and finally to 0.1 mM CaSO_4_ for 1 min (Duan et al., 2007). The ^15^N influx rate was calculated according to methods described by Tang et al. (2012).

### Assessment of Dry Weight, Total N and Metal Ion Accumulation

To investigate the links between *OsNRT2* function and metal ion uptake, we investigated the levels of metal elements using the ICP-OES method in *OsNRT2.1* transgenic lines and Mn elements in *OsNRT2.3a/b* transgenic lines. The creation and identification processes of bO-1, bO-2, bO-8 for *OsNRT2.3b* transgenic lines and aO-1, aO-2 for *OsNRT2.3a* transgenic lines were performed as previously described (Fan et al., 2016).

Fresh WT or transgenic lines were harvested at the rice mature stage (*n* = 4) and heated at 105°C for 30 min. Panicles, flag leaves, second and third leaves, sheaths and stems were then dried for 3 days at 75°C. Rice obtained from hydroponic experiments was divided into shoots and roots only. Dry weights were recorded as biomass values.

Using the Kjeldahl method (Li et al., 2006), total N accumulation was assessed in the different plant areas through multiplying the N concentration by the corresponding biomass. Dried samples were wet-digested in concentrated HNO_3_ at 120°C until no brown nitrogen oxide gas was emitted. When the samples became transparent, they were further digested with HClO_4_ at 180°C. Samples were then diluted with ultrapure water and the concentrations of metal elements in the digestates were analyzed using ICP-OES (iCAP 6300).

### Statistical Analysis

All data were analyzed using the Tukey’s test of one-way analysis of variance (ANOVA). Statistically significant differences at the *p* < 0.05 level (one-way ANOVA) between transgenic and WT and/or between other treatments were assessed. All statistical evaluations were performed using IBM SPSS Statistics version 20 software (SPSS Inc., Chicago, IL, United States).

## Results

### Assessment of Mn Absorption Under Different N Treatments

Wild type rice seedlings were planted under different conditions of N supply. Symptomatically, the roots of rice seedlings were better in 0.5 mM NO_3_^-^ than in 0.5 mM MH_4_^+^, 2.5 mM NO_3_^-^ and 2.5 mM NH_4_^+^ conditions (**Figure 1A**). Statistical analysis showed that the dry weights of the plant roots in 0.5 mM NO_3_^-^ condition were significantly increased (**Figure 1B**). Low concentration NO_3_^-^ could promote root elongation and increase root hairs (Kiba and Krapp, 2016). For the whole plant, the dry weight was best in 2.5 mM N, with no differences between 2.5 mM NO_3_^-^ and 2.5 mM NH_4_^+^ observed (**Figure 1C**). Next, the total N in rice seedlings was investigated. Total N content in rice seedlings with 2.5 mM N supply was higher than that of the 0.5 mM N supply (**Supplementary Figure S1**). Rice seedlings planted in 2.5 mM NH_4_^+^ nutrient solution, displayed the best outcome (**Supplementary Figure S1**).

Simultaneously, the Mn concentration and content of rice roots in 0.5 mM NO_3_^-^ was found to increase more than other conditions. However, shoots were lowest in 2.5 mM NH_4_^+^ solution (**Figures 1D,E**). The Mn content of rice seedlings in NO_3_^-^ solution was higher than in NH_4_^+^ using the same N concentrations (**Figure 1F**). In addition, the expression of the nitrate transporters *OsNRT2.1/OsNRT2.3* were up-regulated by external NO_3_^-^ and the expression of *OsNAR2.1* increased in 0.5 mM NO_3_^-^/2.5 mM NO_3_^-^ compared to NH_4_^+^ treatments in the different tissues (**Supplementary Figures S2A–C**). The expression of Mn transporters *OsNRAMP3/OsNRAMP5/OsNRAMP6* also increased following NO_3_^-^ treatment compared with NH4^+^ treatment. Taken together, these results reveal that both Mn uptake and *OsNRAMP3*/*OsNRAMP5*/*OsNRAMP6* expression are increased by NO_3_^-^. Therefore, NO_3_^-^ positively regulates the absorption of Mn in rice.

### Assessment of the Expression Patterns of OsNRT2s and OsNAR2.1 in the Roots of Transgenic Lines

Firstly, transgenic lines were identified by southern blot analysis and RT-PCR. The data showed that three transgenic lines were one copy insertions and *OsNRT2.1* was overexpressed to approximately five-fold higher mRNA levels in roots and shoots under normal N conditions (1.25 mM NH_4_NO_3_ supply) (**Supplementary Figure S4** and Chen et al., 2016a). WT and transgenic *OsNRT2.1* lines were planted in 0.5 mM NO_3_^-^/NH_4_^+^ nutrition solution, respectively. RT-PCR was performed to confirm the gene expression patterns of the two families of NO_3_^-^ transporters in WT and *OsNRT2.1* transgenic lines under different N supplies. *OsActin* was used as a reference gene for comparison. Total RNA was extracted from the rice roots of the different lines. Under conditions of low concentration (0.5 mM) of NH_4_^+^ and NO_3_^-^, the expression of *OsNRT2.1* in transgenic lines increased 4.5-fold and 5.7-fold, compared to WT (**Figures 2A,B**). No differences in the relative expression of other NO_3_^-^ transporters *OsNRT2.3a/OsNRT2.4* between transgenic and WT lines or between NO_3_^-^ and NH_4_^+^ treatments were observed (**Figures 2A,B**). However, the expression levels of *OsNAR2.1* increased approximately 80% in transgenic lines in 0.5 mM NO_3_^-^, but not in 0.5 mM NH_4_^+^ (**Figures 2A,B**). The total N content of the three transgenic lines was higher than WT in the roots and the shoots under 0.5 mM NO_3_^-^ conditions, with no differences in the NH_4_^+^ solution observed (**Supplementary Figure S5**). Taken together, these results show that *OsNRT2.1* expression is enhanced in the transgenic rice. In addition, the expression of *OsNRT2.1* and *OsNAR2.1* is enhanced in all transgenic lines, allowing an efficient transfer of NO_3_^-^ in 0.5 mM NO_3_^-^ conditions.

### NH4^+^ and NO_3_^-^ Influx Rates in WT and *OsNRT2.1* Transgenic

**FIGURE 3:**
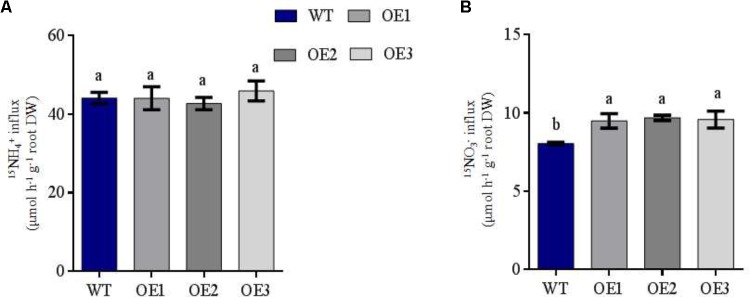
NH_4_^+^ and NO_3_^-^ influx rates of WT and transgenic plants measured using ^15^N-enriched sources. WT and transgenic rice seedlings were grown in IRRI nutrient solution containing 1 mM NH_4_^+^ for 2 weeks and N-starved for 3 days. NO_3_^-^ and NH_4_^+^ influx rates were then measured at **(A)** 0.5 mM ^15^NH_4_^+^ and **(B)** 0.5 mM ^15^NO_3_^-^ for 5 min. DW: dry weight. Error bars: standard error (*n* = 4 plants). Significant differences between transgenic and WT lines are indicated by different letters (*P* < 0.05, one-way ANOVA).

To confirm the influence of *OsNRT2.1* on high-affinity root NO_3_^-^ influx into intact plants, short-term nitrate absorption was assessed by transferring all the lines to either 0.5 mM ^15^NH_4_^+^ or 0.5 mM ^15^NO_3_^-^ for 5 min. Under 0.5 mM ^15^NH_4_^+^ treatment condition, the three transgenic lines displayed no significant differences to WT (**Figure 3A**). However, *OsNRT2.1* transgenic lines were enhanced by 19% compared to WT during NO_3_^-^ influx (**Figure 3B**). In addition, the effects of overexpression on rice growth under different forms of N supply were studied by comparing the total N concentration and content in different parts of the rice plants. The total N of the transgenic lines did not significantly differ in the roots and shoots compared to WT lines in 0.5 mM NH_4_^+^ solution (**Supplementary Figures S5A,B**). However, the total N content of the roots and shoots of the transgenic rice plants was enhanced by 97% and 36%, respectively, compared to WT lines in 0.5 mM NO_3_^-^ conditions (**Supplementary Figure S5E**). Total N concentrations in the shoots did not differ from WT (**Supplementary Figure S5D**). These results show that the overexpression of the high-affinity nitrate transporter *OsNRT2.1* improves NO_3_^-^ uptake in 0.5 mM NO_3_^-^, compared to WT.

### Mn Concentration of Shoots and Roots of Transgenic Plants Under Different N Treatments

**FIGURE 4:**
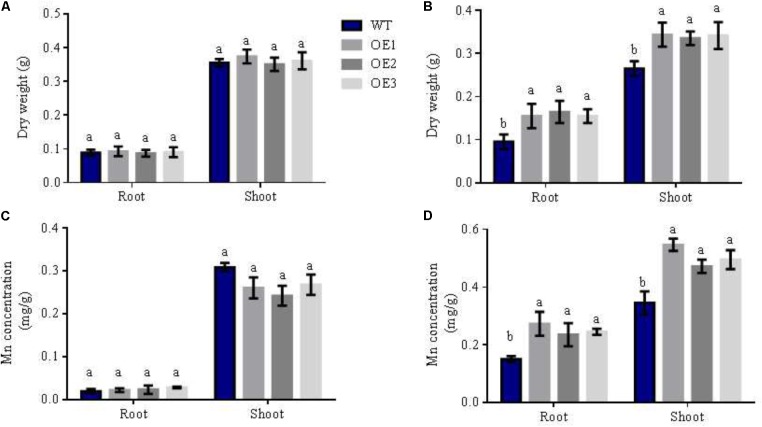
Mn concentration of different lines in different N forms. WT and transgenic lines were treated as in **Figure 2**. Dry weight and Mn concentrations of shoots and roots in WT and *OsNRT2.1* lines with **(A,C)** 0.5 mM NH_4_^+^, **(B,D)** 0.5 mM NO_3_^-^. Error bars: standard error (*n* = 4 plants). Data represent mean differences between plant lines under different N sources. Letters indicate statistically significant differences (*P* < 0.05, one-way ANOVA).

The transferability of Mn is weak. From hydroponic experiments (**Figure 4**), we tested the Mn concentration of shoots and roots in rice when planted in different nutritive forms of N. We found that the dry weight of roots and shoots increased by 66 and 29%, respectively, in transgenic lines relative to WT lines in 0.5 mM NO_3_^-^ solution (**Figure 4B**). However, dry weights did not significantly differ in 0.5 mM NH_4_^+^ (**Figure 4A**). Simultaneously, Mn concentrations of roots and shoots in the overexpression lines were also enhanced by 43% and 47%, respectively, in 0.5 mM NO_3_^-^ solution, but not in 0.5 mM NH_4_^+^ (**Figures 4C,D**). From **Figure 4** and **Supplementary Figure S3**, we reasoned that this was due to the *OsNRT2.1* gene transferring NO_3_^-^ into the rice, increasing total N, Mn uptake and accumulation in 0.5 mM NO_3_^-^ condition. These results indicate that Mn assimilation by *OsNRT2.1* is NO_3_^-^ uptake dependent, and that the overexpression of *OsNRT2.1* does not only increase NO_3_^-^ uptake to enhance total N, but also promotes Mn absorption in rice in low NO_3_^-^ condition.

### Effects of Different Irrigation Conditions on N and Mn Concentrations in Grain

**FIGURE 5:**
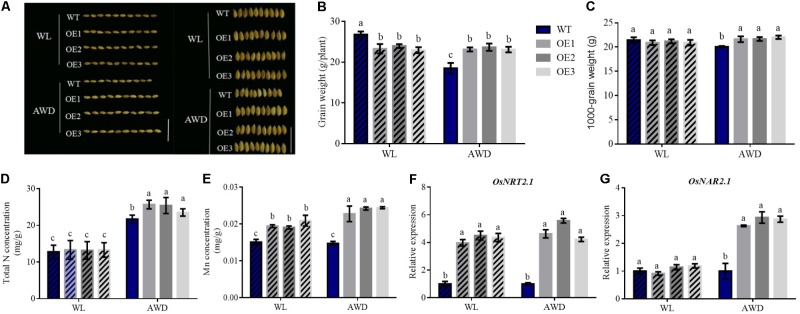
Effects of different irrigation conditions on N and Mn concentrations in grain. **(A)** Seed morphology images, **(B)** Grain weights, **(C)** 1000-grain weight, **(D)** total N concentration, **(E)** Mn concentration of seeds in WT and transgenic lines under different field experiments. Total RNA was extracted from the culm of WT and overexpression lines and the results of qPCR under different irrigation conditions was used to assess the relative expression of **(F)** *OsNRT2.1* and **(G)** *OsNAR2.1*. Error bars: standard error (*n* = 4 plants). WL, waterlogged; AWD, alternating wet and dry conditions. Different letters indicate a significant difference between WT and overexpression lines or in differing irrigation conditions of all lines (*P* < 0.05, one-way ANOVA). Bars = 1 cm.

Rice typically grows in anaerobic flooded fields, which exist mainly in the form of NH_4_^+^-N. Conversely, NO_3_^-^ is present mainly in aerobic uplands (Stitt, 1999). To simulate hydroponic conditions in the presence of different N treatments, we designed a field experiment under different irrigation conditions and investigated *OsNRT2.1* function on rice grains in the field. From the assessment of seed morphology, seeds of WT under alternating wet and dry (AWD) condition were shorter than other seeds (**Figure 5A** and **Supplementary Figure S6**). Compared to other field treatments, we found that the grain weight of WT in AWD condition was approximately 31% lower than waterlogged (WL) condition, with no differences in the transgenic lines observed (**Figure 5B**).

In addition, no evident differences in all lines in WL condition were observed. However, the grain weight of transgenic rice plants was approximately 26% higher compared to WT weights in AWD condition (**Figure 5A**). The 1000-grain weight displayed a similar pattern to the grain weights (**Figure 5B**). We also tested the total N concentration of the seeds under different field treatments. Interestingly, WT and transgenic lines were higher in AWD condition compared to waterlogged condition, and the total N concentration of the transgenic seeds also increased by 15% compared to WT in the AWD field (**Figure 5C**). However, the total N concentration of the husk in the overexpression lines was lower than that of WT in the AWD field, whilst no differences in all lines from the WL field were observed (**Supplementary Figure S7A**). As higher levels of N were transferred into the seeds of transgenic lines in the AWD field, their seed weights were higher than WT.

Simultaneously, the Mn concentrations in the seeds of transgenic lines in AWD condition were enhanced when compared to WL condition. No differences were observed for the different field conditions in WT lines (**Figure 5D**). In addition, the husk of grain displayed similar results in terms of Mn concentrations (**Supplementary Figure S7B**). The concentration of Fe and Mg in seeds and husk appeared to vary irregularly (**Supplementary Figure S7**). This presented the unity of the Mn element.

These results demonstrate that rice planted in AWD condition displays higher total N and Mn concentrations in grain, particularly for *OsNRT2.1* transgenic lines. We extracted total RNA from the culm of all lines planted in the two types of irrigated field. From **Figures 5F,G**, the relative expression of *OsNRT2.1* in transgenic lines was higher than WT lines in WL and AWD conditions. However, *OsNAR2.1* expression was enhanced 2.8-fold only in AWD field relative to WT. Therefore, the soil of AWD primarily existed in NO_3_^-^ form to enhance NO_3_^-^ uptake through increased *OsNRT2.1* expression, leading to the induction of *OsNAR2.1* expression. As the relative expression of *OsNRT2.1* and *OsNAR2.1* increase following AWD treatment, NO_3_^-^ uptake may further improve Mn uptake compared to the WL field.

### Assessment of the Expression of Related Genes, Total N and Mn Accumulation During Maturity Stages in AWD Conditions

To understand mechanism(s) of how *OsNRT2.1* improves total N and Mn accumulation at the mature stage in AWD field, we extracted total RNA from the different areas of rice (**Supplementary Figure S9**) and assessed the expression of *OsNRT2.1, OsNAR2.1*, and Mn transporters-*OsNRAMP3, OsNRAMP5*, and *OsNRAMP6* (Yamaji et al., 2013a; Yang et al., 2014; Peris-Peris et al., 2017).

**FIGURE 6:**
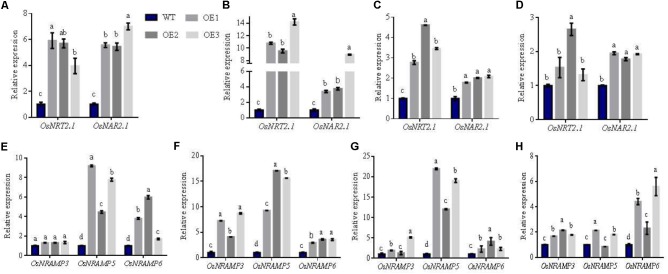
Relative expression of related genes in different arears of WT and transgenic plants in AWD fields. Total RNA was isolated from **(A,E)** panicle-neck, **(B,F)** flag leaves, **(C,G)** flag leaves sheaths, and **(D,H)** Node I of WT and transgenic lines. Error bars: standard error (*n* = 4 plants). Different letters indicate a significant difference between WT and overexpression lines (*P* < 0.05, one-way ANOVA).

From **Figure 6**, the expression of the related nitrate genes-*OsNRT2.1* and *OsNAR2.1* in the three transgenic lines were higher in the panicle-neck, flag leaves, flag leaves sheaths and node I compared to WT rice. The panicle-neck connects vegetative and reproductive organs. Flag leaves are functional leaves for transferring nutrients. Studies have reported that high-affinity nitrate *OsNRT2.1* requires its partner protein *OsNAR2.1* to transfer nitrate (Feng et al., 2011; Yan et al., 2011; Tang et al., 2012). Accordingly, the expression of *OsNRT2.1* and its partner protein *OsNAR2.1* increased in the Panicle-neck and in the functional leaves at maturity. NO_3_^-^ was transferred to the panicle to enhance total N accumulation in seeds, and further improve grain yields.

**FIGURE 7:**
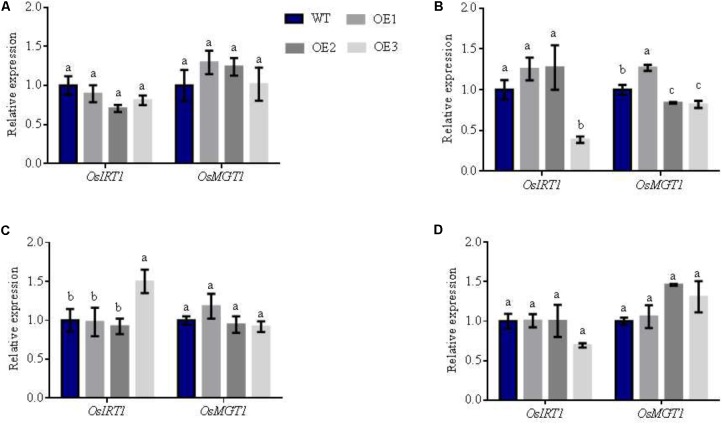
Relative expression of other related metal genes in the different lines under AWD conditions. Total RNA was isolated from **(A)** panicle-neck, **(B)** flag leaves, **(C)** flag leaves sheaths and **(D)** Node I of WT and overexpression lines. Error bars: standard error (*n* = 4 plants). Different letters indicate a significant difference between WT and transgenic lines (*P* < 0.05, one-way ANOVA).

Interestingly, we found that the expression of the Mn transporters: *OsNRAMP3, OsNRAMP5*, and *OsNRAMP6* were also upregulated in transgenic lines. These genes displayed similar expression patterns to NO_3_^-^ transporters (**Figures 6E–G**). In particular, the expression of *OsNRAMP3* and *OsNRAMP6* in the transgenic lines increased by 87 and 311% in comparison to WT in the node I, respectively (**Figure 6H**). Node I represents the junction of the vascular system connecting the leaves, stems and panicles. Therefore, Mn transporter genes-*OsNRAMP3* and *OsNRAMP6* preferentially transport Mn to flag leaves and the panicle during the late stages of plant growth in rice. We found that the biomass of transgenic and WT lines displayed no significant differences at maturity (**Supplementary Figure S10A**). The NO_3_^-^ concentrations of the different plant areas (except for leaves in the overexpression lines) were higher than WT (**Supplementary Figure S10D**). However, total N accumulation did not differ in various parts of the plants, and Mn showed an irregular trend without flag leaves (**Supplementary Figure S10**). These results suggest that total N and Mn are transferred to grains from vegetative organs at maturity. We further assessed Fe and Mg content in various parts of the different lines, in which we observed no differences (**Supplementary Figure S11**). When the relative expression of *OsIRT1* and *OsMGT1* that represent Fe and Mg related genes (Lee and An, 2009; Chen Z.C. et al., 2017) were analyzed, the expression patterns were also inconsistent in diverse areas of the transgenic rice plants (**Figure 7**).

Taken together, these data suggest that the improvement of Mn concentration in *OsNRT2.1* lines was due to the increased expression of Mn transporters, but no effects on other metal elements were observed.

## Discussion

Nitrate and Mn are essential nutrients in plants, and it has been reported that Mn deficiency decreases N uptake and metabolism (Gong et al., 2011). Excessive NO_3_^-^ was shown to enhance Cd uptake in *Thlaspi caerulescens* (Xie et al., 2009) and wheat (Li et al., 2011). In addition, crosstalk between mineral elements exists. Wang et al. (2015) reported that Al improves Mn uptake and accumulation in rice roots, However, these tactics do not enhance the security of crops for human consumption because they do not increase the accumulation of beneficial elements in plant. In this study, the main objective was to investigate how interactions between Mn and NO_3_^-^ influence rice growth and nutrient accumulation in roots, leaves tissues and grain.

We found that NO_3_^-^ improves Mn uptake in rice (**Figure 2**). When Mn concentrations were assessed in *OsNRT2.1/OsNRT2.3a/b* transgenic lines planted in normal field which was WL condition, respectively (**Supplementary Figure S3**), we observed increased Mn in the seeds and husk of *OsNRT2.1* (**Supplementary Figures S3A,D**), but no enhanced uptake in *OsNRT2.3a/b* transgenic lines (**Supplementary Figures S3B,C,E,F**). Given these data, we investigated the pattern of Mn accumulation in *OsNRT2.1* transgenic lines in further detail, under differing conditions of N supply and field conditions. This information is important as rice typically grows in anaerobic flooded fields, in which N exists mainly in the form of NH_4_^+^, as opposed to aerobic uplands where the major form of N is NO_3_^-^ (Stitt, 1999). We found that *OsNRT2.1*-regulates NO_3_^-^ uptake in roots, which in turn increases Mn root entry. This increases the Mn concentration in rice grain in the presence of low concentrations of NO_3_^-^ and under AWD condition. Thus, enhancing *NRT2.1*-mediated NO_3_^-^ uptake represents an attractive mechanism of increasing Mn accumulation in food.

### Effects of NO_3_^-^ and NH_4_^+^ Nutrition on Mn Accumulation

We demonstrate that NO_3_^-^ nutrition promotes Mn assimilation in plants to higher levels than NH_4_^+^ nutrition (**Figure 1**). Thus Mn availability in nutrient solutions is influenced by the type of N-nutrient treatments. In 0.5 mM NH_4_^+^, Mn uptake in the roots did not differ in *OsNRT2.1* transgenic lines (**Figure 4C**). However, Mn uptake drastically increased in the transgenic lines in 0.5 mM NO_3_^-^ (**Supplementary Figure S5** and **Figure 4D**). It was recently shown that NO_3_^-^ uptake induces external alkalization, reducing Fe/Mn concentrations by enhancing the levels of H_2_O_2_ in rice (Chen et al., 2018). In this study, we performed hydroponic experiments in MES buffered nutrient medium (to control pH) and nutrient treatments were replaced every 2 days. Furthermore, the Mn content in grain from field experiments should not be influenced by pH as the rhizosphere ranges from pH 5.5 to 6.0 in paddy soil (Pan et al., 2016). Thus, any effects of soil alkalization were excluded. We thus hypothesize that NO_3_^-^ upregulates the expression of Mn transporters, including *OsNRAMP3, OsNRAMP5, and OsNRAMP6* to increase Mn uptake and accumulation (**Figure 1** and **Supplementary Figure S2**). We verified the expression of *OsNRT2.1* and *OsNAR2.1* under NH_4_^+^ and NO_3_^-^ conditions in transgenic *OsNRT2.1* lines and found that *OsNRT2.1* was unaffected by the different N forms. However, the expression of its partner protein *OsNAR2.1* was significantly up-regulated in 0.5 mM NO_3_^-^ (**Figure 2**). The *OsNRT2.1* lines could still promote NO_3_^-^ uptake (**Figure 3**) into the different tissues compared to WT plants (**Supplementary Figure S10D**). Thus, the up-regulation of *OsNAR2.1* expression in NO_3_^-^ condition (**Figure 2B**) promotes NO_3_^-^ uptake in transgenic plants compared with WT (**Supplementary Figure S10D**). The observation that the upregulation of *OsNRT2.1/OsNAR2.1* is favorable to the transport of NO_3_^-^ in plants and improves rice yield, is consistent with our previous findings (Chen et al., 2016b; Chen J.G. et al., 2017). As we did not observe enhanced expression of either *OsNRT2.3a* or *OsNRT2.4* lines in NO_3_^-^ condition, we speculate that the regulation of *OsNAR2.1* differs from other *OsNRT2* genes according to the plant NO_3_^-^ content (**Supplementary Figure S2**, Yan et al., 2011; Wei et al., 2018).

### AWD vs. WL Conditions in WT vs. Transgenic Lines

In field experiments, the grain weight of WT lines under AWD condition decreased by 31% compared to WL condition. The NO_3_^-^ concentrations in *OsNRT2.1* transgenic lines also differed across plant areas in AWD condition and which were higher than WT lines (**Supplementary Figure S10D**). However, the total N concentration did not significantly differ across the lines (**Supplementary Figure S10C**). The total N of seeds in transgenic lines increased compared to WT (**Figure 5**). Thus, the overexpression of *OsNRT2.1* improves NO_3_^-^ uptake and assimilation efficiency to increase N accumulation in grain, leading to enhanced grain yields. In hydroponic experiments, the overexpression of *OsNRT2.1* also enhanced NO_3_^-^ uptake (**Figure 3B**) and N accumulation (**Figure 5D**), maintaining plant grain yields in WL condition. This is because in AWD condition, a high concentration of dissolved oxygen is present, which can influence nitrification by nitrifying bacterial, or chemical oxidation for the conversion of NH_4_^+^ to NO_3_^-^ at the root surface (Li et al., 2008; Steffens et al., 2011). Thus, under AWD condition, NO_3_^-^ plays an important role in N accumulation and contributes to enhanced grain yields. However, for WT plants, the capacity to uptake NO_3_^-^ is limited; and thus, grain yields are dramatically reduced. This observed loss of grain in WT type rice can likely be explained by a multitude of mechanisms.

Surprisingly, we found that under AWD, Mn in the grain of transgenic plants was greatly increased compared to WL condition, but in WT rice, no changes were evident (**Figure 5E**). In addition, in WL condition, Mn levels also increased in the transgenic lines compared to WT (**Figure 5E**). We observed no differences in Mn concentrations in other parts of the plant under AWD condition (**Supplementary Figure S10**). Thus, higher levels of Mn were transported to grain and accumulated (**Figure 5**). This explains the improvement in seed quality, emergence, and seeding growth observed, as the positive effects of Mn on these processes is well documented (Dimkpa and Bindraban, 2016). The seeds of *OsNRT2.1* overexpression lines not only increased in their total N accumulation, but enhanced Mn content was also observed (**Figure 5**). The length/width of these seeds were also better than WT (**Figure 5A** and **Supplementary Figure S6**), demonstrating that Mn plays an important role in increasing crop nutritional quality, crop yield and biomass production. Other metal elements such as Mg and Fe were not influenced by *OsNRT2.1* overexpression (**Supplementary Figures S8, S11**).

### Enhanced Expression of Mn Transporters Explains Enhanced Mn Uptake in Transgenic Lines

We verified gene expression profiles in the organs responsible for grain filling and discovered that the expression of *OsNRT2.1* and *OsNAR2.1* were enhanced in the panicle-neck, flag leaves and sheaths (**Figure 6A**). In the same plant areas, the expression of *OsNRAMP5* and *OsNRAMP6* increased in the *OsNRT2.1* lines. Interestingly, *OsNRAMP3/6* expression was enhanced in node I (**Figure 6B**). The expression of related genes involved in Mg and Fe uptake were also altered by *OsNRT2.1* overexpression (**Figure 7**). It is understood that node I is a junction of vasculatures that link leaves, stems and panicles and so is important for the transport of nutrient elements into grain (Yamaji and Ma, 2009, 2014; Yamaji et al., 2013a). Transporters responsible for the delivery of minerals into seeds have been reported, including *OsYSL16* for Cu (Zheng et al., 2012), *OsHMA2* for Zn and Cd (Yamaji et al., 2013b) and *AtNIP6;1* that is expressed in the node region for B distribution (Tanaka et al., 2008). Accordingly, the majority of these genes are also strongly expressed in node I (Tanaka et al., 2008; Yamaji and Ma, 2009, 2014; Zheng et al., 2012; Yamaji et al., 2013a).

## Conclusion

Taken together, we show that AWD treatment can induce the expression of NO_3_^-^ and Mn transporters in grain filling organs which increases the accumulation of N and Mn in grain. NO_3_^-^ uptake in *OsNRT2.1* transgenic lines can improve Mn accumulation, however, the Mn concentration does not increase in the seeds and husk of *OsNRT2.3a/b* overexpression lines, which also display increased NO_3_^-^ uptake compared to WT lines (Fan et al., 2016). Thus, the mechanism(s) linking NO_3_^-^ and Mn in *OsNRT2.1* overexpressing plants differ from other *OsNRT2* overexpression lines and is worthy of further investigation. From our findings, we propose a new application to improve both N and water efficiency in agricultural systems and demonstrate how high *OsNRT2.1* expression improves Mn content in rice grain.

## Author Contributions

BiL, JC, and XF conceived the study, analyzed the data, and drafted the manuscript. BiL, LZ, and SL cultivated the rice materials and collected the rice samples. LZ, BL, and HL extracted RNA and performed the qRT-PCR experiments. BL and LZ participated in field and material management. BiL and JC conducted the statistical analysis of raw data. XF, GX, and GY revised the manuscript. All authors read and approved the final manuscript.

## Conflict of Interest Statement

The authors declare that the research was conducted in the absence of any commercial or financial relationships that could be construed as a potential conflict of interest.

## Supplementary Material

FIGURE S1 Assessment of the total N-content under different N treatments. **(A)** total N concentration, **(B)** total N content of roots and shoots from different N treatments, **(C)** total N content of whole plants. 0.5/2.5A: 0.5 mM/2.5 mM NH_4_^+^ as an N source; 0.5/2.5 N: 0.5/2.5 mM NO_3_^-^ as an N source. Error bars: standard error (*n* = 4 plants). Different letters indicate a significant difference between N treatments (*P* < 0.05, one-way ANOVA).

FIGURE S2 Expression pattern of NO_3_^-^ transporters and Mn transporters under different N treatments; total RNA was isolated from WT rice supplied with 0.5/2.5N: 0.5/2.5 mM NO_3_^-^ and 0.5/2.5 A:0.5/2.5 mM NH_4_^+^ as an N source for 2 weeks. **(A)** relative expression of *OsNRT2.1*/*OsNRT2.3*/*OsNAR2.1* and **(D)** *OsNRAMP3*/*OsNRAMP5*/*OsNRAMP6* in leaves; **(B)** relative expression of *OsNRT2.1*/*OsNRT2.3*/*OsNAR2.1* and **(E)** *OsNRAMP3*/*OsNRAMP5*/*OsNRAMP6* in sheath; **(C)** relative expression of *OsNRT2.1*/*OsNRT2.3*/*OsNAR2.1* and **(F)** *OsNRAMP3*/*OsNRAMP5*/*OsNRAMP6* in roots. Error bars: standard error (*n* = 4 plants). Different letters indicate a significant difference between different N treatments (*P* < 0.05, one-way ANOVA).

FIGURE S3 Mn concentration in seeds and husk of *OsNRT2.1*/*OsNRT2.3b* overexpression lines. Mn concentrations in seeds of **(A)** *OsNRT2.1* overexpression lines, **(B)** *OsNRT2.3b* overexpression lines and **(C)** *OsNRT2.3a* overexpression lines. Mn concentration in husk of **(D)** *OsNRT2.1* overexpression lines, **(E)** *OsNRT2.3b* overexpression lines and **(F)** *OsNRT2.3a* overexpression lines. b-O1/2/8: three *OsNRT2.3b* overexpression lines; a-O1/2: two *OsNRT2.3a* overexpression lines. Error bars: standard error (*n* = 4 plants). Different letters indicate a significant difference between N treatments (*P* < 0.05, one-way ANOVA).

FIGURE S4 Identification of transgenic lines. **(A)** Southern blot of genomic DNA isolated from WT and transgenic plants. Hybridization was performed using a hygromycin gene probe. P, positive control; M, marker. Extraction of total RNA from roots and shoots of WT and transgenic lines and qRT-PCR results under. **(B)** M: DNA molecular-weight marker II, DIG – labeled; P: positive controls. Error bars: standard error (*n* = 4 plants). Different letters indicate a significant difference between N treatments (*P* < 0.05, one-way ANOVA).

FIGURE S5 Comparison of total N/Mn concentrations and content of transgenic plants at different nitrogen supply levels. **(A–C)** Under 0.5 mM NH_4_^+^ treatments, **(A)** total N concentration, **(B)** total N content and **(C)** Mn content of roots and shoots. **(D–F)** Under 0.5 mM NO_3_^-^ treatments, **(D)** total N concentration, **(E)** total N content and **(F)** Mn content of roots and shoots. Error bars: standard error (*n* = 4 plants). Different letters indicate a significant difference between WT and overexpression lines (*P* < 0.05, one-way ANOVA).

FIGURE S6 Assessment of the length and width of seeds in different lines under WL and AWD treatments. **(A)** Seeds lengths (mm), **(B)** seed widths (mm). Error bars: standard error (*n* = 4 plants), 15 repeats. Different letters indicate a significant difference between WT and overexpression lines (*P* < 0.05, one-way ANOVA).

FIGURE S7 Effects of different irrigation conditions on Mn concentrations in rice husk. Under WL and AWD, **(A)** total N concentration and **(B)** Mn concentration of rice husk were assessed. Error bars: standard error (*n* = 4 plants). Different letters indicate a significant difference between the irrigation conditions of all lines (*P* < 0.05, one-way ANOVA).

FIGURE S8 Effects of different irrigation conditions on other elements in rice seeds. Under WL and AWD, Fe and Mg concentrations of husk **(A,B)** and seeds **(C,D)**. Error bars: standard error (*n* = 4 plants). Different letters indicate a significant difference between WT and overexpression lines in different irrigation conditions (*P* < 0.05, one-way ANOVA).

FIGURE S9 Diagram of RNA sampling in WT and transgenic plants.

FIGURE S10 Effect of transgenic lines on total N/Mn content in vegetative organs under AWD conditions. **(A)** Dry weight of different parts in all lines, **(B)** Total N concentration, **(C)** Total N content, **(D)** NO_3_^-^ concentration, **(E)** Manganese concentration, and **(F)** Manganese content from different parts of all lines. Error bars: standard error (*n* = 4 plants). Other leaves: second and third leaves. Different letters indicate a significant difference between WT and overexpression lines (*P* < 0.05, one-way ANOVA).

FIGURE S11 Concentration of other elements in different parts of transgenic lines under AWD conditions. **(A)** Mg concentration, **(C)** Mg content, **(B)** Fe concentration, **(D)** Fe content. Error bars: standard error (*n* = 4 plants). Different letters indicate a significant difference between WT and overexpression lines (*P* < 0.05, one-way ANOVA).

FIGURE S12 Correlation analysis between expression of *OsNRT2.1* and total nitrogen/nitrate concentration in flag leaves of wt and transgenic lines. **(A)** Linear Analysis of relative expression of *OsNRT2.1* and total N concentration. **(B)** Linear Analysis of relative expression of *OsNRT2.1* and nitrate concentration.

TABLE S1 Primers used to amplify the *OsNRT2.1* open reading frame.

TABLE S2 Primers used for quantitative real-time polymerase chain reaction.
