# Supplementary figures and images for: Overexpression of a High-Affinity Nitrate Transporter OsNRT2.1 Increases Yield and Manganese Accumulation in Rice Under Alternating Wet and Dry Condition
Source: Front Plant Sci. 2018 Aug 15;9:1192. doi: 10.3389/fpls.2018.01192 (PMC6104626; doi:10.3389/fpls.2018.01192)

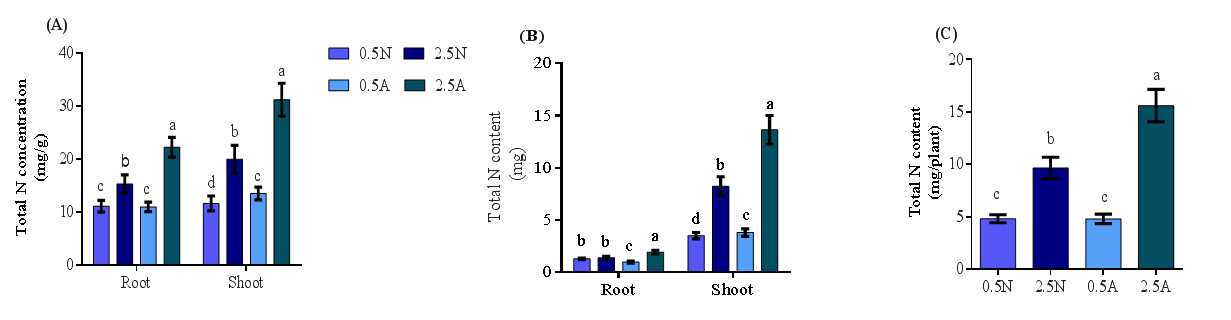

Supplement: FIGURE S1 — Assessment of the total N-content under different N treatments. (A) total N concentration, (B) total N content of roots and shoots from different N treatments, (C) total N content of whole plants. 0.5/2.5A: 0.5 mM/2.5 mM NH4+ as an N source; 0.5/2.5 N: 0.5/2.5 mM NO3- as an N source. Error bars: standard error (n = 4 plants). Different letters indicate a significant difference between N treatments (P < 0.05, one-way ANOVA). [file Image_1.JPEG]

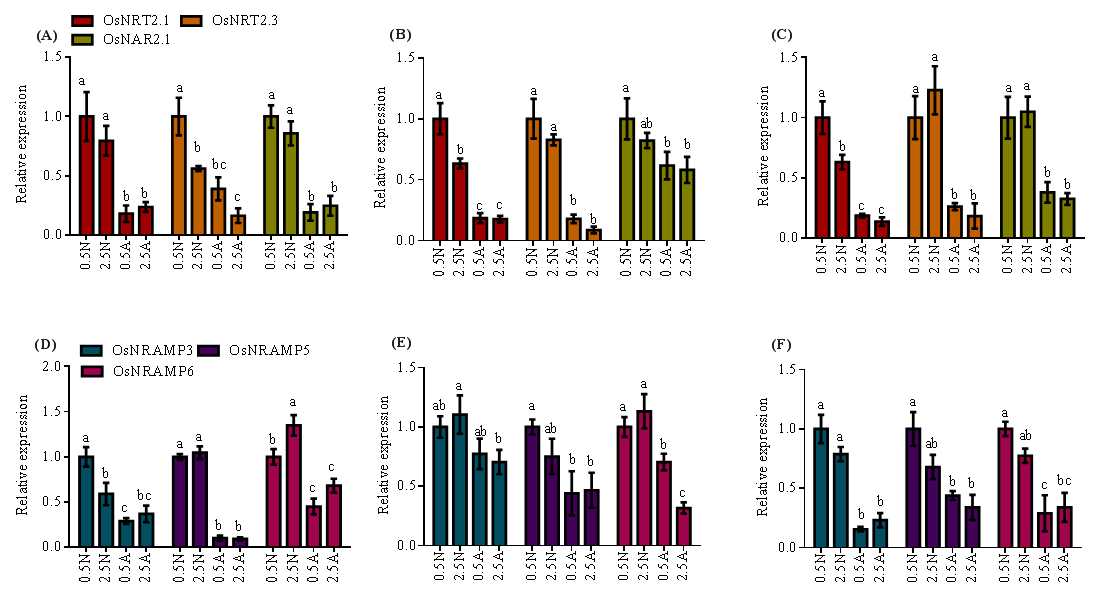

Supplement: FIGURE S2 — Expression pattern of NO3- transporters and Mn transporters under different N treatments; total RNA was isolated from WT rice supplied with 0.5/2.5N: 0.5/2.5 mM NO3- and 0.5/2.5 A:0.5/2.5 mM NH4+ as an N source for 2 weeks. (A) relative expression of OsNRT2.1/OsNRT2.3/OsNAR2.1 and (D) OsNRAMP3/OsNRAMP5/OsNRAMP6 in leaves; (B) relative expression of OsNRT2.1/OsNRT2.3/OsNAR2.1 and (E) OsNRAMP3/OsNRAMP5/OsNRAMP6 in sheath; (C) relative expression of OsNRT2.1/OsNRT2.3/OsNAR2.1 and (F) OsNRAMP3/OsNRAMP5/OsNRAMP6 in roots. Error bars: standard error (n = 4 plants). Different letters indicate a significant difference between different N treatments (P < 0.05, one-way ANOVA). [file Image_2.JPEG]

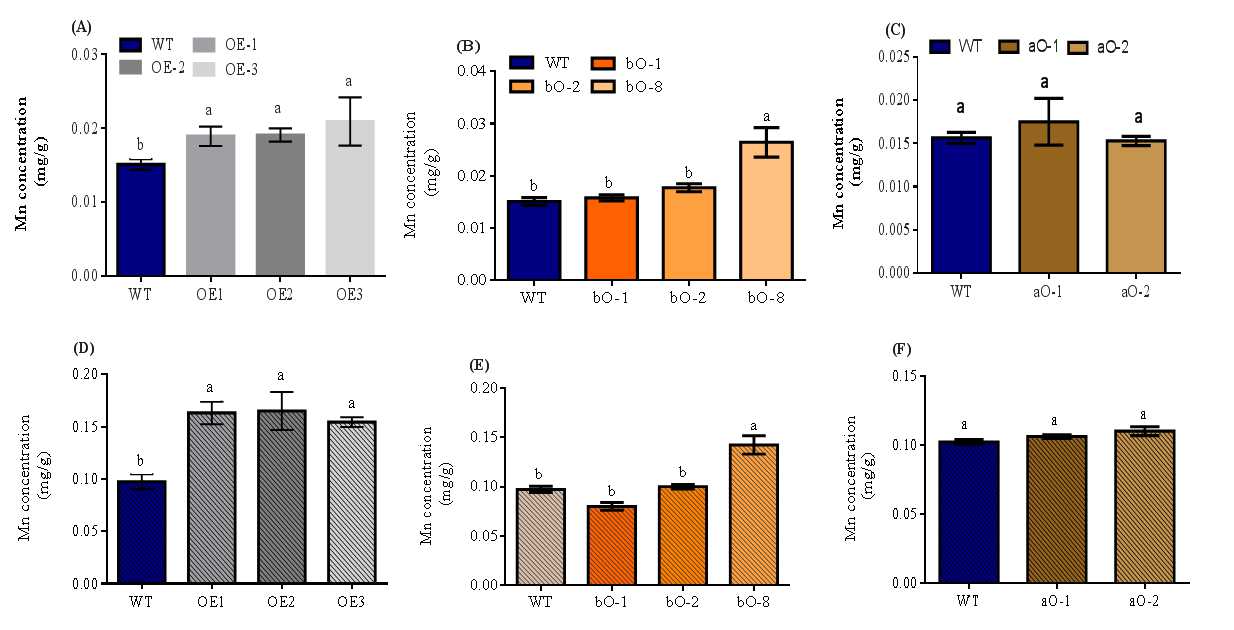

Supplement: FIGURE S3 — Mn concentration in seeds and husk of OsNRT2.1/OsNRT2.3b overexpression lines. Mn concentrations in seeds of (A) OsNRT2.1 overexpression lines, (B) OsNRT2.3b overexpression lines and (C) OsNRT2.3a overexpression lines. Mn concentration in husk of (D) OsNRT2.1 overexpression lines, (E) OsNRT2.3b overexpression lines and (F) OsNRT2.3a overexpression lines. b-O1/2/8: three OsNRT2.3b overexpression lines; a-O1/2: two OsNRT2.3a overexpression lines. Error bars: standard error (n = 4 plants). Different letters indicate a significant difference between N treatments (P < 0.05, one-way ANOVA). [file Image_3.JPEG]

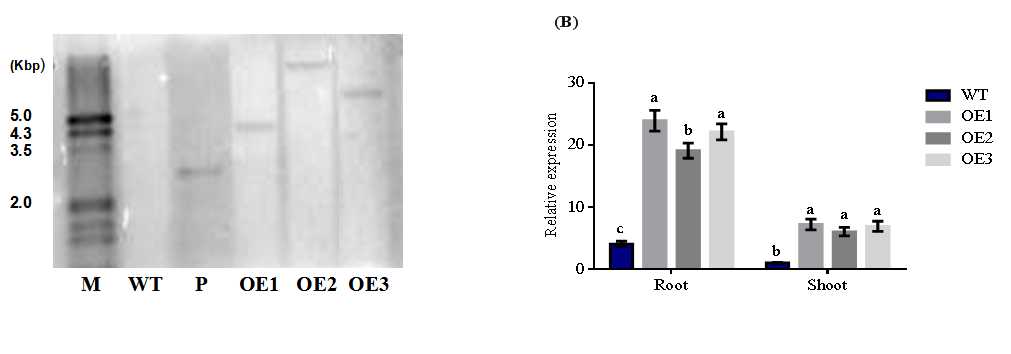

Supplement: FIGURE S4 — Identification of transgenic lines. (A) Southern blot of genomic DNA isolated from WT and transgenic plants. Hybridization was performed using a hygromycin gene probe. P, positive control; M, marker. Extraction of total RNA from roots and shoots of WT and transgenic lines and qRT-PCR results under. (B) M: DNA molecular-weight marker II, DIG – labeled; P: positive controls. Error bars: standard error (n = 4 plants). Different letters indicate a significant difference between N treatments (P < 0.05, one-way ANOVA). [file Image_4.JPEG]

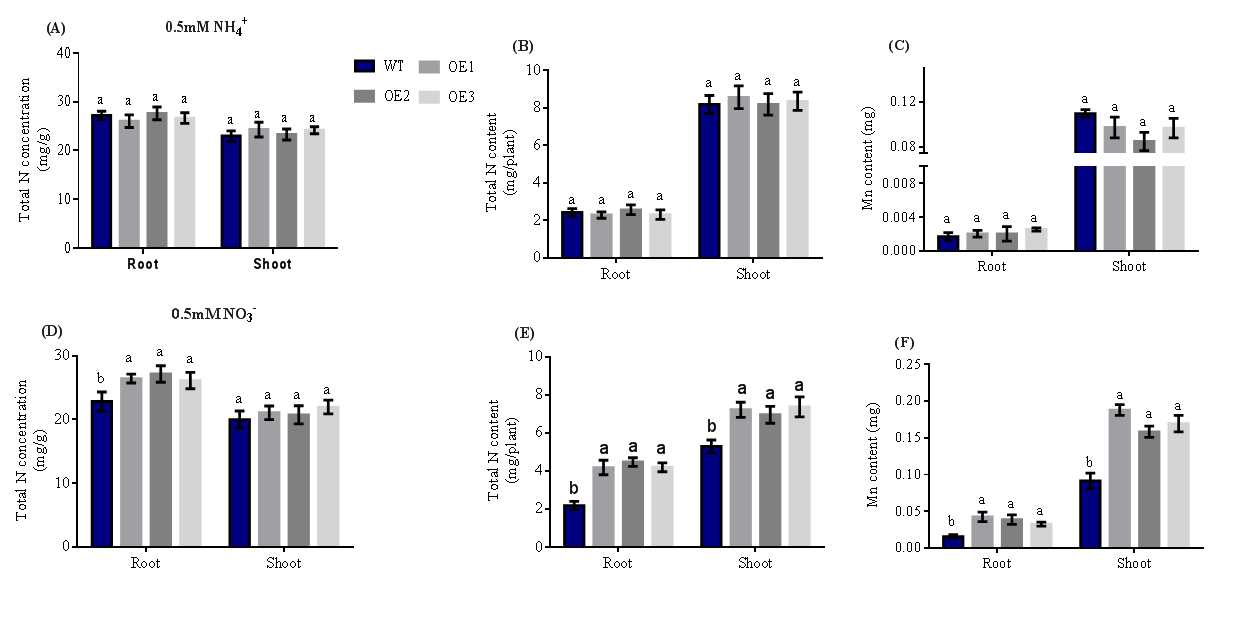

Supplement: FIGURE S5 — Comparison of total N/Mn concentrations and content of transgenic plants at different nitrogen supply levels. (A–C) Under 0.5 mM NH4+ treatments, (A) total N concentration, (B) total N content and (C) Mn content of roots and shoots. (D–F) Under 0.5 mM NO3- treatments, (D) total N concentration, (E) total N content and (F) Mn content of roots and shoots. Error bars: standard error (n = 4 plants). Different letters indicate a significant difference between WT and overexpression lines (P < 0.05, one-way ANOVA). [file Image_5.JPEG]

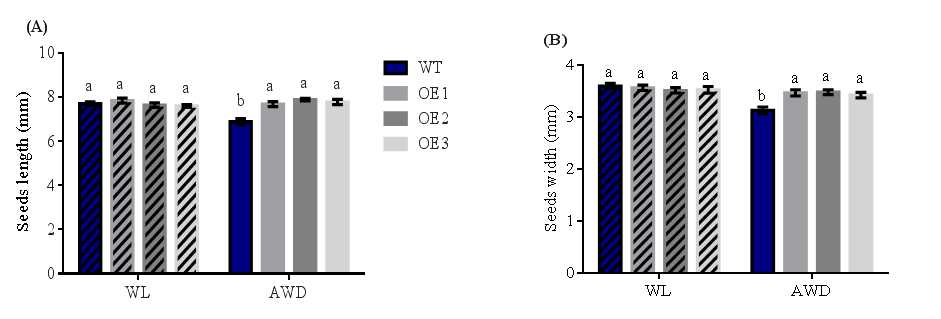

Supplement: FIGURE S6 — Assessment of the length and width of seeds in different lines under WL and AWD treatments. (A) Seeds lengths (mm), (B) seed widths (mm). Error bars: standard error (n = 4 plants), 15 repeats. Different letters indicate a significant difference between WT and overexpression lines (P < 0.05, one-way ANOVA). [file Image_6.JPEG]

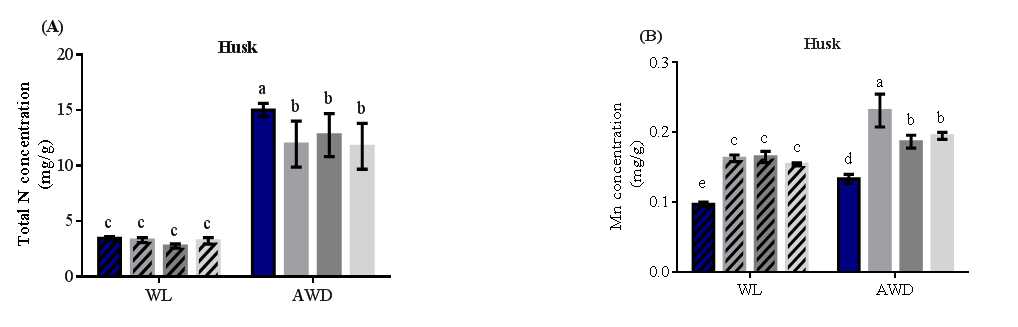

Supplement: FIGURE S7 — Effects of different irrigation conditions on Mn concentrations in rice husk. Under WL and AWD, (A) total N concentration and (B) Mn concentration of rice husk were assessed. Error bars: standard error (n = 4 plants). Different letters indicate a significant difference between the irrigation conditions of all lines (P < 0.05, one-way ANOVA). [file Image_7.JPEG]

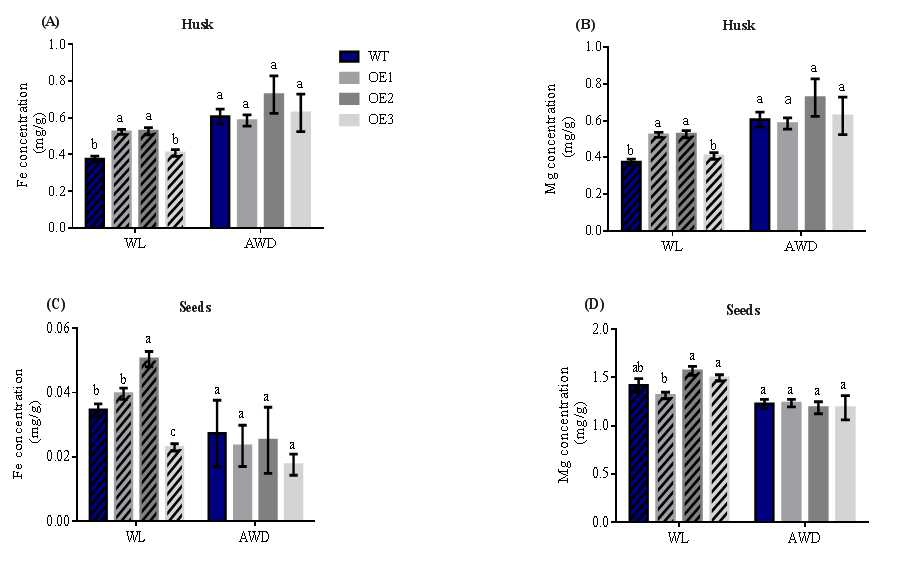

Supplement: FIGURE S8 — Effects of different irrigation conditions on other elements in rice seeds. Under WL and AWD, Fe and Mg concentrations of husk (A,B) and seeds (C,D). Error bars: standard error (n = 4 plants). Different letters indicate a significant difference between WT and overexpression lines in different irrigation conditions (P < 0.05, one-way ANOVA). [file Image_8.JPEG]

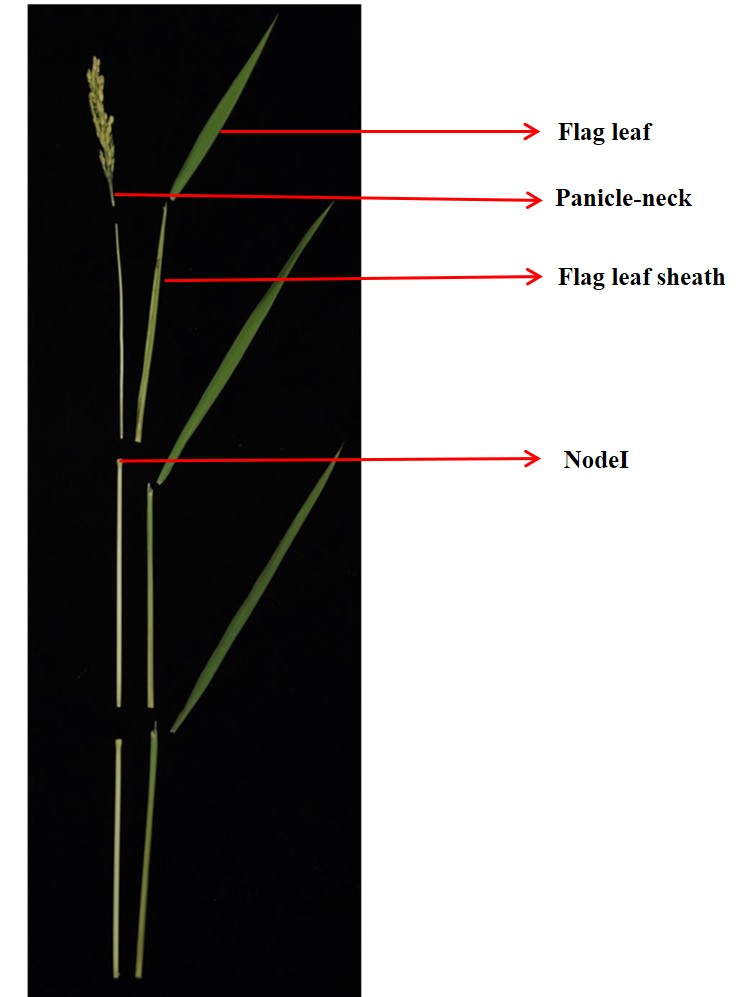

Supplement: FIGURE S9 — Diagram of RNA sampling in WT and transgenic plants. [file Image_9.JPEG]

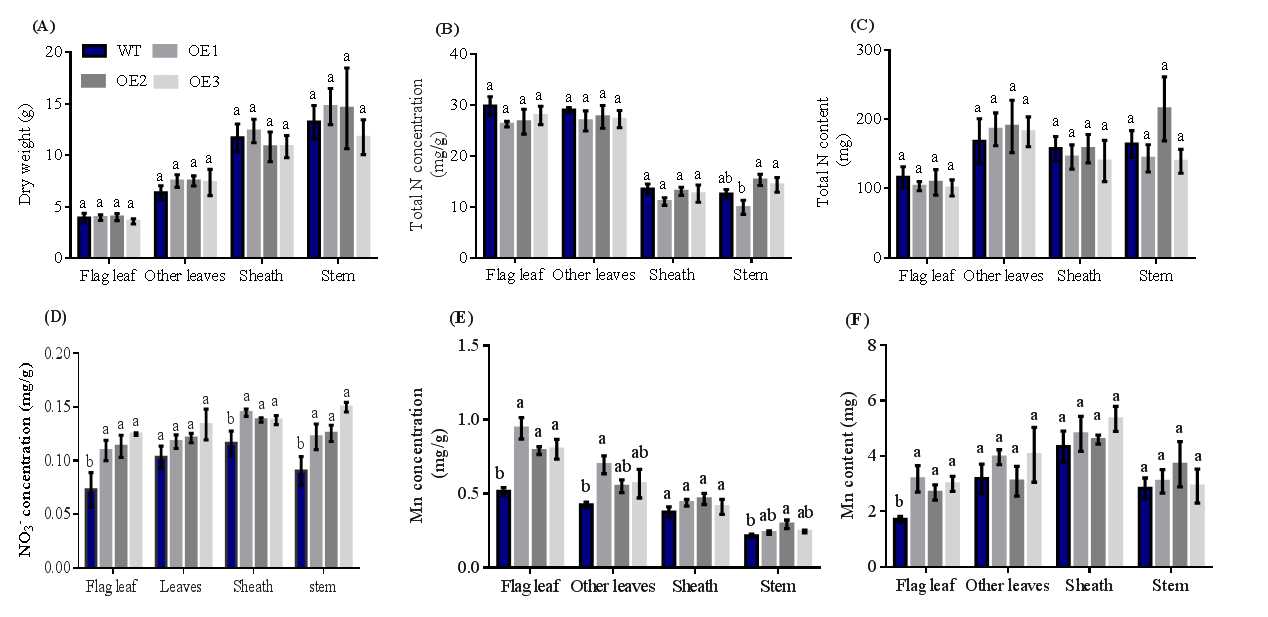

Supplement: FIGURE S10 — Effect of transgenic lines on total N/Mn content in vegetative organs under AWD conditions. (A) Dry weight of different parts in all lines, (B) Total N concentration, (C) Total N content, (D) NO3- concentration, (E) Manganese concentration, and (F) Manganese content from different parts of all lines. Error bars: standard error (n = 4 plants). Other leaves: second and third leaves. Different letters indicate a significant difference between WT and overexpression lines (P < 0.05, one-way ANOVA). [file Image_10.JPEG]

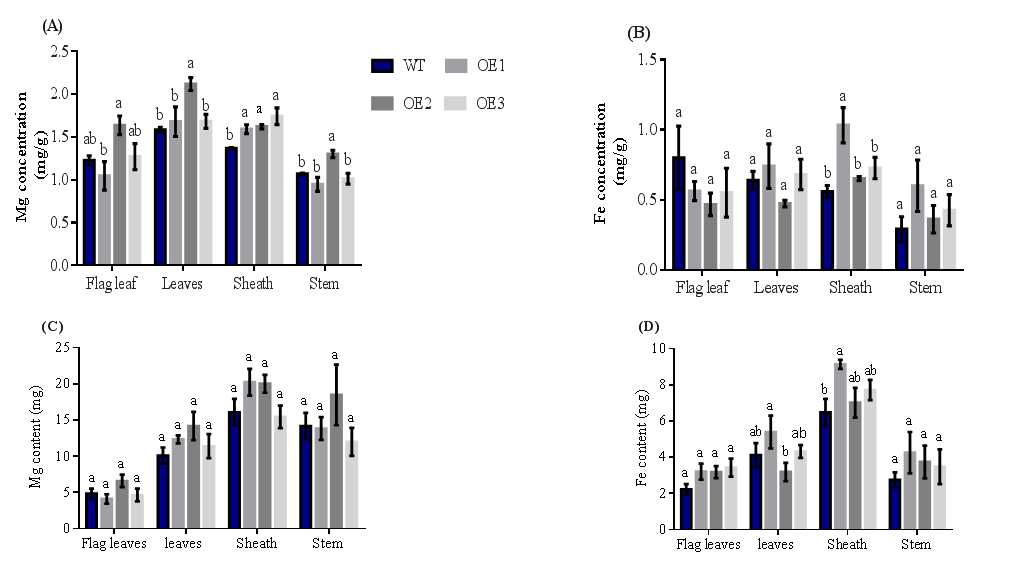

Supplement: FIGURE S11 — Concentration of other elements in different parts of transgenic lines under AWD conditions. (A) Mg concentration, (C) Mg content, (B) Fe concentration, (D) Fe content. Error bars: standard error (n = 4 plants). Different letters indicate a significant difference between WT and overexpression lines (P < 0.05, one-way ANOVA). [file Image_11.JPEG]

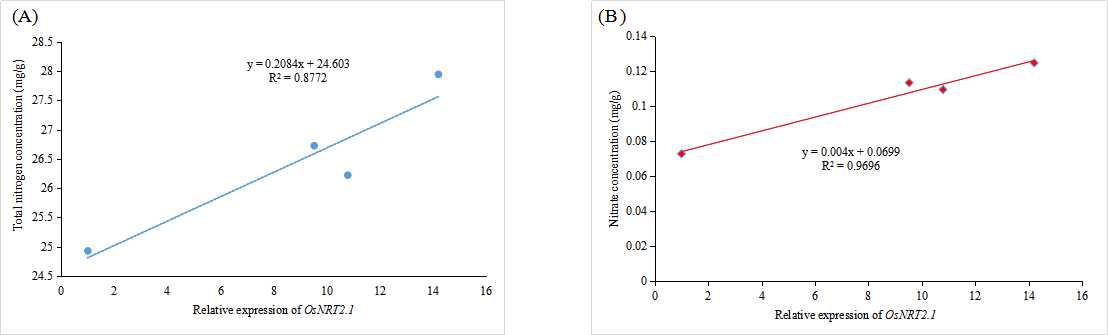

Supplement: FIGURE S12 — Correlation analysis between expression of OsNRT2.1 and total nitrogen/nitrate concentration in flag leaves of wt and transgenic lines. (A) Linear Analysis of relative expression of OsNRT2.1 and total N concentration. (B) Linear Analysis of relative expression of OsNRT2.1 and nitrate concentration. [file Image_12.JPEG]
