# Supplementary material for: Overexpression of a High-Affinity Nitrate Transporter OsNRT2.1 Increases Yield and Manganese Accumulation in Rice Under Alternating Wet and Dry Condition
Source: Front Plant Sci. 2018 Aug 15;9:1192. doi: 10.3389/fpls.2018.01192 (PMC6104626; doi:10.3389/fpls.2018.01192)
Supplement: TABLE S1 — Primers used to amplify the OsNRT2.1 open reading frame. [file Table_1.PDF]

Gene name	Primer	Sequence
OsNRT2.1	5' (F)	CCAAGGTACCATGGACTCGTCGACGGTGGGC
	5' (R)	GGCCACTAGTTTAGGCGTGCTCCGGCGAG
