# Supplementary material for: Overexpression of a High-Affinity Nitrate Transporter OsNRT2.1 Increases Yield and Manganese Accumulation in Rice Under Alternating Wet and Dry Condition
Source: Front Plant Sci. 2018 Aug 15;9:1192. doi: 10.3389/fpls.2018.01192 (PMC6104626; doi:10.3389/fpls.2018.01192)
Supplement: TABLE S2 — Primers used for quantitative real-time polymerase chain reaction. [file Table_2.PDF]

Gene name	Forward primer (5'to 3')	Reverse primer (5'to3')
OsActin	CAACACCCCTGCTATGTACG	CATCACCAGAGTCCAACACAA
OsNRT2.1	CCATCATCCGCGACAACC	TGGCGAGCCTGGAGAAG
OsNRT2.3a	GCCATCCACAAGATCGGTAG	TGTGGAGCTTCCCGTAGTTG
OsNRT2.4	CCCTTCGTCTGCAAAAGGT	TACCTGGACCCGCTGAAGAA
OsNAR2.1	GTCGTCGAGAAGCGCAAGA	GTCCACTGAAGCTGCGAACTT
OsNRAMP3	TCAGCAGCGAACTGCTTCTGATCT	ATCAGCTGGCTAACTCTTTGGGCT
OsNRAMP5	GTCGGAGCCGTTCGTTTAT	GGCTCTGCCCTGAATTATGA
OsNRAMP6	GCTCAAAGCCTCGAAATCAT	TGGCGTGGAAGAGAATTTTA
OsIRT1	AGGTCGGTGCTCGTCTTCT	TGTCCCTGTACACCCTGGTC
OsMGT1	GGCGCGTGCAGAAGATTAGGG	CGCGTATTCACGGATATGGTACAGGG
